# Ethnic disparities in patients with early-onset Alzheimer’s disease (EOAD)

**DOI:** 10.3389/fneur.2025.1643514

**Published:** 2025-12-10

**Authors:** Meredith Yang, Suemin Jasmine Yoon, Kristen Taruc, Phyllis Timpo, James Mastrianni, Kaitlin Seibert

**Affiliations:** 1 Pritzker School of Medicine, University of Chicago, Chicago, IL, United States; 2 Feinberg School of Medicine, Northwestern University, Chicago, IL, United States; 3 Department of Neurology, University of Chicago Biological Sciences Division, Chicago, IL, United States; 4 Healthy Aging & Alzheimer’s Research Care (HAARC) Center, University of Chicago, Chicago, IL, United States; 5 Center for Brain Health, Cleveland Clinic, Cleveland, OH, United States

**Keywords:** early-onset Alzheimer’s disease (EOAD), young onset dementia (YOD), Alzhemer’s dementia, ethnic disparities, healthcare access

## Abstract

**Introduction:**

Ethnic disparities in Early-onset Alzheimer’s Disease (EOAD) have not been fully characterized.

**Methods:**

We retrospectively identified 81 EOAD patients February 2017–February 2022.

**Results:**

53 (65.4%) identified as White and 28 (34.6%) identified as Black. Age at diagnosis (years) was (57.3 ± 5.24) in Black patients compared to (56.3 ± 4.96) in White patients. Time to diagnosis (years) was longer in Black patients (3.39 ± 1.85) compared with White patients (2.64 ± 1.75). Black patients had lower MOCA scores (12.17 ± 6.9) compared with White patients (14.9 ± 5.8). An amnestic profile was more common in Black patients (19 or 67.9%) than non-amnestic presentations (9 or 32.1%). Non-amnestic presentations were more common in White patients, with 9 (17%) patients with PCA, 8 (15%) with fAD and 8 (15%) with l-PPA.

**Discussion:**

In our cohort, Black patients with EOAD are on average older, more advanced and experienced longer delays in diagnosis than their White counterparts.

## Introduction

Early-onset Alzheimer’s Disease (EOAD) affects patients younger than age 65 and may present with non-amnestic features. Black adults are up to twice as likely as White adults to develop Alzheimer disease (AD) (1, 2). In addition, studies suggest that Black patients with dementia not only experience greater delays to diagnosis but they are often undertreated, which may reflect disparities in access to healthcare, relative lack of AD awareness, and systemic distrust (3–6). Whereas ethnoracial disparities have been reported for typical late-onset Alzheimer’s Disease (LOAD), there is a relative paucity of similar studies for diverse patients with EOAD.

Most studies on atypical presentations of AD, including EOAD, focus on white populations (7, 8) Compared to typical AD, little is known about the differences in pathogenesis, genetic diagnostic biomarker profile, and natural history, particularly among Black patients (9). Interestingly, Black and Latino AD patients may have longer survival compared with white AD patients (10). Clinically, Black participants with dementia may have higher neuropsychiatric symptoms/severity and functional dependence as compared to White patients with dementia (11). Studies suggest that the underlying pathogenesis of cognitive impairment in AD may be different in Black patients compared to White patients (12). Despite disproportionately higher rates of AD among people wo identify as Black, a recent studies found lower odds of amyloid PET positivity among Black and Hispanic patients compared with White patients (13, 14).

Only one study specifically characterized biomarker differences between ethnicities, finding that Black patients had lower levels of total and phosphorylated tau (t-tau and p-tau), and amyloid-beta (Ab) levels in their cerebrospinal fluid (CSF) when compared with non-Hispanic White people (15). Another study also found lower levels of CSF t-tau and p-tau among Black patients, though it was found to be perhaps a function of ApoE4 status (16).

Pathologically, Black patients with AD are more likely to have mixed pathology than White patients with AD, which may at least partially account for the variability in biomarkers (17). Further study of biomarker and clinicodemographic data may be the key to providing culturally competent care for Black patients with AD.

As a tertiary care center with a large Black population on the South Side of Chicago, we sought to compare several aspects of the utilization of medical services by patients, the perceived clinical features of the disease at presentation, diagnostic testing, and clinical features of disease among Black and White patients diagnosed with EOAD. We also aimed to identify differences in diagnostic tests ordered and obtained to help inform and identity potential barriers in diagnosis in the Black EOAD population.

## Materials and methods

The study was reviewed and approved by the Institutional Review Board of the affiliated academic medical center.

### Study sample

We retrospectively identified EOAD patients from 02/01/2017 to 02/01/2022 by extracting all patients who presented to a single academic memory center and were diagnosed with Early Onset Alzheimer’s Dementia by a board-certified behavioral neurologist. All patients had at least met criteria for probable Alzheimer’s disease based on McKhann et al. (18) 2011 guidelines. We compared data on ethnicity, level of education, Montreal Cognitive Assessment (MoCA) scores and clinical phenotype through chart review of electronic health records. MoCA scores were available for the majority of our patients and is known to be more sensitive in detecting early cognitive impairment compared to the Mini-Mental State Examiniation (MMSE) score, which can be simpler and easier to administer. MMSE was collected for patients for which MoCA scores were not available. We defined EOAD as patients with AD with onset of symptoms younger than 65 years of age. We also included patients that expressed symptoms before 65, yet presented to clinic after age 65. This was elicited by asking the patient and their accompanying family members to report at what age symptoms first onset and an estimate of age at onset was then made. We also collected data on frequency of diagnostic tests ordered and completed through further chart review of the same patients.

Non-black and non-white patients constituted less than 5% of the study sample and thus were excluded. Only data available in electronic medical record was included.

Subtypes of clinical phenotypes were made based on clinical syndrome at the discretion of a board certified behavioral neurologist.

### Statistical analysis

Descriptive statistics were generated for demographic variables. These included mean with standard deviation or median with interquartile range (IQR) for continuous and categorical variables. The independent sample t-test and the Fisher’s exact test were used for continuous variables and categorical variables, respectively. All statistical tests were two-sided with a *p*-value < 0.05 as statistically significant. Analyses were performed using STATA/SE software version 18 (StataCorp LP, Texas, USA).

## Results

### Demographics

We identified 81 EOAD patients with 28 Black patients and 53 White patients (Table 1). Of the 81 participants, 47 or 58% of our sample was female. Interestingly, more than two-thirds of our total Black patients were female (19 or 67.8%). The average length of education obtained was 15.3 years. The average age of disease onset was 56.7 years, with Black patients reporting slightly higher mean age of onset than White patients (57.3 ± 5.24 versus 56.3 ± 4.96). The average years from onset to diagnosis was 2.9 years. Mean years from onset to diagnosis was higher in Black patients versus White patients (3.39 years±1.85 compared to 2.64 years±1.75). Positive family history was present in 34.6% of our sample.

**Table 1:** Demographic characteristics.

Variable, *n*	Overall	Black patients	White patients	*p*-value
	(*n* = 81)	(*n* = 28)	(*n* = 53)	
Female sex (%)	47 (58%)(*n* = 51)	19 (67.8%)(*n* = 14)	28 (52%)(*n* = 37)	
Average years of education (SD)	15.27 (2.57)	15 (2.18)	15.38 (2.7)	*p* < 0.6446
Average age of onset (SD)	56.7 (5.05)	57.3 (5.24)	56.3 (4.96)	*p* < 0.4002
Average years from onset to diagnosis (SD)	2.90 (1.81)	3.39 (1.85)	2.64 (1.75)	*p* < 0.0761
Positive family history (%)	28 (34.6%)(*n* = 65)	10 (35.7%)(*n* = 23)	18 (34.0%)(*n* = 42)	*p* = 0.496
Average MoCA score (SD)	13.95 (6.30)(*n* = 8)	12.17 (6.9)(*n* = 3)	14.9 (5.8)(*n* = 5)	*p* < 0.0923
Average MMSE score (SD)	17.75 (7.00)	14.3 (7.02)	19.8 (6.87)	*p* < 0.3210
**MoCA domains average scores (SD) *n* = 62**				
Visuospatial executive	2.06 (1.49)	2.13 (1.42)	2.02 (1.5)	*p* < 0.7811
Naming	2.29 (0.89)	2.09 (0.97)	2.4 (0.8)	*p* < 0.1952
Attention	3.17 (2.11)	2.72 (2.14)	3.43 (2.09)	*p* < 0.2168
Language	1.32 (1.18)	1.18 (1.26)	1.4 (1.15)	*p* < 0.4922
Abstraction	1.21 (0.79)	0.86 (0.83)	1.4 (0.70)	*p* < 0.0096
Delayed recall	0.20 (0.48)	0.05 (0.21)	0.3 (0.56)	*p* < 0.047
Delayed recall with category cues (*n* = 56)	0.73 (0.94)	0.45 (0.69)	0.89 (1.04)	*p* < 0.096
Delayed recall with multiple choice (*n* = 56)	1.45 (1.26)	1.45 (1.31)	1.44 (1.25)	*p* < 0.988
Orientation	3.77 (1.80)	3.40 (1.87)	3.98 (1.76)	*p* < 0.2408
**Test results**				
Average T_tau (SD) *n* = 32	624.6 (283.23)	615.35 (249.74)	626.00 (291.8)	*p* < 0.945
Average P_tau *n* = 32	89.80 (31.87)	95.8 (19.85)	88.95 (33.41)	*p* < 0.695
Average A_beta *n* = 32	397.30 (148.96)	426.2 (66.8)	393.18 (157.6)	*p* < 0.685
*Other*	*Total*	*Black*	*White*	
	(*n* = 81)	(*n* = 28)	(*n* = 53)	
Mortality at 5 years (*n* = 81)	48 (60%)(*n* = 80)	17 (60%)(*n* = 27)	31 (58%)(*n* = 53)	1.000
Is the patient adherent to acetylcholinesterase inhibitor?	65 (81%)(*n* = 37)	21 (78%)(*n* = 11)	44 (83%)(*n* = 26)	0.5610.296
Is the patient adherent to antipsychotics?	33 (89%)(*n* = 80)	11 (100%)(*n* = 28)	22 (84.6%)(*n* = 53)	
Is the patient accompanied at last visit?	80	28 (100%)	52 (98%)	

### MoCA scores

65 of our 81 patients (80%) completed the Montreal Cognitive Assessment (MoCA) at presentation. All participants scored less than or equal to 26, the recommended cutoff that supports cognitive impairment (19); the overall mean MoCA score was 13.95 ± 6.3. Average MoCA scores were lower among Black patients (12.17 ± 6.9) compared to White patients (14.9 ± 5.8). This was also reflected in MMSE scores (*n* = 8), with Black patients scoring on average lower than White patients (14.3 ± 7.02 versus 19.8 ± 6.87). However, neither of these results are statistically significant.

Mean scores in separate MoCA items were lower across all domains for Black patients compared to White patients. Black patients scored lower in abstraction (mean score of 0.86 ± 0.83 out of a total score of 2), compared to White patients (mean score of 1.4 ± 0.70) (*p* < 0.05). Black patients also scored lower in delayed free recall, with a mean score of 0.05 ± 0.21 compared to White patients (0.3 ± 0.56, *p* < 0.05).

### Clinical phenotype

Black patients presented most commonly with an amnestic phenotype. Within our cohort, 19 of 28 (68%) had an amnestic presentation at initial evaluation, five (18%) presented with prominent language impairment (logopenic primary progressive aphasia), one (4%) had a presentation characteristic of a dysexecutive variant of Alzheimer’s disease, and two (7%) had cortical visual symptoms suggestive of posterior cortical atrophy (PCA) (see Table 2).

**Table 2:** Clinical phenotype, by ethnicity.

Clinical phenotype of AD	Total (%)	Black patients (%)	White patients (%)
Amnestic	45 (56%)	19 (68%)	26 (49%)
PCA	11 (14%)	2 (7%)	9 (17%)
Language (PPA)	13 (16%)	5 (18%)	8 (15%)
Executive dysfunction	9 (11%)	1 (4%)	8 (15%)
Indeterminate	3 (4%)	1 (4%)	2 (4%)

**Figure 1:**
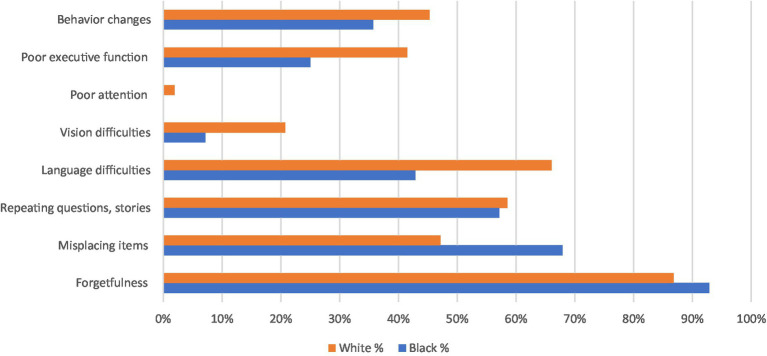
Presenting symptoms, by ethnicity.

These findings are supported by Figure 1, in which we report the most frequent presenting symptom in the initial clinic visit by ethnicity. Black patients more frequently reported forgetfulness, misplacing items, repeating questions and stories compared to other more atypical symptoms such as language and vision difficulties, or behavioral changes.

### Diagnostic testing completion rates

In general, Black patients obtained and completed fewer diagnostic tests than White patients (Table 3). Brain MRI, CSF testing for Alzheimer’s Disease biomarkers, fluorodeoxyglucose-positron emission tomography (FDG-PET) brain scan, single photon emission computed tomography (SPECT) brain scan, comprehensive neuropsychological assessment, genetic testing, and brain amyloid-PET scan usage were compared among the two cohorts. We found that White patients, compared with Black patients, completed significantly more CSF testing (53% vs. 14%, *p* < 0.001), FDG-PET imaging (40% vs. 18%, *p* < 0.05), and neuropsychological assessments (75% vs. 25%, *p* < 0.001). A similar trend was found in the way clinicians ordered diagnostic tests. CSF testing was ordered in 53% of White patients, compared to just 14% of Black patients (*p* < 0.001) (Table 3). Similarly, neuropsychological testing was much more frequently ordered in White patients compared to Black patients (83% vs. 39%) (*p* < 0.001). In contrast, a brain MRI was ordered at similarly high rates in both Black and White patients (93% compared to 92%).

**Table 3:** Diagnostic tests completed and ordered, by ethnicity.

	Total *n* = 81	%	Black patients *n* = 28	%	White patients *n* = 53	%	*p* value
Testing							
Completed MRI	72	89%	24	86%	48	91%	0.712
Completed CSF testing	32	40%	4	14%	28	53%	*p* < 0.001
Completed FDG-PET	26	32%	5	18%	21	40%	*p* < 0.05
Completed SPECT	30	37%	8	29%	22	42%	0.335
Completed Neuropsychological testing	47	58%	7	25%	40	75%	*p* < 0.001
Completed genetic testing	21	26%	7	25%	14	26%	1.000
Completed amyloid PET testing	7	9%	3	11%	4	8%	0.688
Ordered or not?							
MRI ordered	75	93%	26	93%	49	92%	1.000
CSF testing ordered	32	40%	4	14%	28	53%	*p* < 0.001
FDG-PET testing ordered	28	35%	6	21%	22	42%	0.088
SPECT testing ordered	32	40%	10	36%	22	42%	0.642
Neuropsychological testing ordered	55	68%	11	39%	44	83%	*p* < 0.000
Genetic testing ordered	37	46%	13	46%	24	45%	1.000
Amyloid PET test ordered	11	14%	5	18%	6	11%	0.500

### Diagnostic testing results

CSF results were available for 32 of our 81 patients (Table 1). There were no statistically significant differences in Black vs. White patients’ CSF concentrations of t-tau (615.35 ± 249.74 vs. 626.00 ± 291.8), p-tau (95.8 ± 19.85 vs. 88.95 ± 33.41), or Aβ42 (426.2 ± 66.8 vs. 393.18 ± 157.6).

## Conclusion and discussion

In our cohort, Black patients with EOAD were, on average, more advanced, and experienced a longer time to diagnosis than their White counterparts. Non-amnestic, atypical presentations were more common in White patients presenting with EOAD, at least in our sample. We also found that Black patients not only completed fewer diagnostic tests that were ordered (specifically CSF testing, FDG-PET imaging, and neuropsychological testing), but as a group, CSF testing and neuropsychological assessments were ordered less frequently in Black patients compared with White patients.

It is important to acknowledge that in our study, Black patients on average experienced a longer period between symptom onset and diagnosis. A 2012 study in a large, Dutch cohort (*n* = 414) found that EOAD patients took an average of 1.6 years longer than LOAD patients to obtain a diagnosis (20). Prolonged time from symptom onset to diagnosis could be due to a number of factors. Atypical, non-amnestic presentation of AD in patients less than 65 years old may be more likely to be misdiagnosed or missed by clinicians.

We also found that Black patients in our cohort trended towards lower scores on the MoCA at presentation. Although this did not reach statistical significance, some of the individual tasks, such as abstractions and delayed recall, did. Does a lower MoCA score in this context necessarily imply greater cognitive impairment? Prior evidence has suggested that older Black participants tend to perform worse on tests of executive function and visuospatial ability, when compared with older White participants, even when controlling for education (21–25).

It is not known whether this finding is also observed in Black patients less than 65 years of age. Interestingly, one of the earliest studies of normative performance on the MoCA in Black participants (1,419 patients with a mean age 49 years) found that total MoCA scores were lower than normative data derived from elderly White participants (26). In the same study, Rossetti et al. (26) found that some domains were especially missed by a large portion of the sample—including delayed free recall and abstraction—as in our cohort. This may suggest that certain items in MoCA have lower psychometric utility for Black participants.

One study using a related cognitive measure, the Mini Mental Status Exam (MMSE), demonstrated a lower score at presentation in Black and Latino patients with AD as compared to White patients with AD, but no appreciable difference among Black and Non-Hispanic White cognitively normal participants (27). The role of cognitive reserve in racially diverse populations also remains unclear. One study found that Black patients have a lower baseline level of cognition but progress more slowly over time; however, additional studies are necessary for further characterization (28).

Lower mean scores specifically in delayed free recall and abstraction items among Black patients are also consistent with our overall finding that amnestic presentations were more common among Black EOAD patients than their White counterparts. This is contrary to our current understanding that atypical, non-amnestic presentations of AD are more characteristic in EOAD cohorts (29). What may account for this difference? This may represent an overall selection bias among our clinic population.

For example, it could be that patients are more likely to present to a memory clinic for symptoms such as forgetfulness than visual difficulties due to wider awareness of the former symptom as a neurological problem. A 2023 study of 4,284 patients from 2008 to 2018 has shown that Black patients who present to memory clinic are more likely to have moderate or severe dementia at their initial visit (30).

Perhaps the most evident disparity was in access to EOAD care, as Black patients completed fewer diagnostic tests than White patients, suggesting additional social determinants of health must be explored in order to further characterize this gap. Providers also ordered relatively lower numbers of lumbar puncture procedures for Black patients by providers, presuming that the provider did not order the study because the patient declined. Lumbar puncture (LP) for CSF testing is one of the most crucial components of EOAD diagnosis, especially when accounting for financial and insurance barriers to care. Existing research has shown, consistent with our study, that Black patients are less likely than White patients to agree to a lumbar puncture (31). A general culture of medical mistrust and the historical context of medical researchers exploiting diverse patients are some of the many reasons patients and their caregivers may be less inclined to agree to an invasive test (32). Further research is needed to determine whether access to non-invasive diagnostic tests such as amyloid PET scans may mitigate diagnostic disparities in EOAD care.

It is also interesting to note that neuropsychological testing was less likely to be completed by Black patients in our cohort. We also find that providers were less likely to order neuropsychological testing for Black patients. Many factors may have contributed to this finding, such insurance barriers, access to transportation or caregiver constraints. Testing itself also takes upwards of 6 h, incurring an additional transportation or time burden for patients that may need transport or work full-time. Coverage for testing is variable for EOAD patients who are younger than 65 years of age. For example, the FDG-PET is not covered by Medicare for EOAD patients, which may partially explain the low completion rate. Provider bias of a patient’s general willingness to pursue such studies may also play a role in the discrepancy between Black and White patients. Further exploration of such barriers is imperative to ensure that the healthcare system is able to deliver uniformly culturally competent care and that all patients have access to early diagnosis and a comprehensive list of treatment options, particularly in the new era of disease-modifying anti-amyloid therapies.

These disparities in care highlight the value of culturally competent and ethnically informed memory care providers in an increasingly demanding medical field. Such awareness is imperative to mitigate rather than exacerbate existing ethnoracial disparities in AD diagnosis and treatment as well as clinical trial enrollment.

### Strengths and limitations

Our study findings are limited by our retrospective and single-center design. It is not known if similar findings are present in other memory clinics. Limitations of electronic medical records should also be noted. While certain information was collected directly through medical records, such as time of diagnosis, we relied on caregivers and family member accounts to obtain an estimated age of symptom onset. Race and ethnicity data was based on self-reported demographic data collected at patient registration, which had specific options to be selected from a list and did not include full aspects of a person’s identity, such as whether they identify as Hispanic or non-hispanic. Additionally, information about the availability of a care partner was unable to be accessed from the medical record. Other social determinants of health such as education level, social and community support, geographical location and financial and insurance status were not able to be reliably collected in our cohort. Finally, our study is limited by a small sample and recruitment from a single medical center, and therefore we are limited by its generalizability to the broader population beyond the Chicago metropolitan area. Although our cohort is small overall, the disparities in care cast on patients with EOAD who identify as Black is important particularly in the era of disease-modifying therapies. EOAD patients are already vulnerable to systemic barriers; identification and awareness of provider biases is key to ensuring access to therapies for all patients.

Further investigation with larger-scale studies that capture the full extent of patients’ identities and barriers to care across institutions and providers is needed to fully characterize accessibility barriers in the EOAD population. Several important components to consider include access to culturally competent dementia specialists, social and community education and support of patients with EOAD, and financial and systemic logistical feasibility of diagnostic tests and continued care. Further characterization of social determinants of health in diverse populations, education of providers and support systems, and reducing systemic barriers to care via needs assessments are the next steps in optimizing early diagnosis and treatment of EOAD.

## Data Availability

The raw data supporting the conclusions of this article can be made available by the authors, without undue reservation.
